# Risk of moderate or severe hypoxic ischemic encephalopathy does not correlate with prenatally known risk factors

**DOI:** 10.1055/a-2638-5623

**Published:** 2025-09-16

**Authors:** Mario Rüdiger, Sven Kehl, Cornelia Wiechers, Angela Kribs, Ulrich Pecks, for the DGPM Research Collaborative

**Affiliations:** 1 39063 Saxonian Center for Feto/Neonatal Health, Faculty of Medicine and University Hospital Carl Gustav Carus, Technische Universität Dresden, Dresden, German Center for Child and Adolescent Health (DZKJ), SaxoChild partner site Dresden/Leipzig, Germany; 2 396211 Department of Obstetrics and Gynecology, LMU University Hospital, LMU Munich, Germany; 3 27203 Department of Neonatology and Interdisciplinary Centre for Cleft Palate and Craniofacial Malformations, University of Tuebingen, Tuebingen, Germany; 4 27182 Department of Pediatrics, Division of Neonatology, Faculty of Medicine and University Hospital Cologne, University of Cologne, Cologne, Germany; 5 9190 Department of Obstetrics, University of Würzburg, Würzburg, Germany

**Keywords:** Perinatal Aspyhxia, Therapeutic Hypothermia, Hypoxic ischemic encephalopathy, prenatal risk

## Abstract

Infants with perinatal asphyxia require immediate support in order to prevent further damage. If asphyxia progresses towards hypoxic ischemic encephalopathy, therapeutic hypothermia (TH) in a specialised NICU is indicated. In order to provide evidence-based recommendations for an appropriate perinatal care structure, data for Germany are needed. German NICUs which offer TH (cooling centres) provided data in order to analyse how many neonates were treated with TH and how many of them were transferred for TH. Furthermore, for transferred infants the level of care of birth hospital was analysed and the rate of neonates with TH per 1,000 deliveries was calculated for each hospital. Data for 1,431 neonates with TH was obtained from 20 cooling centres. The average annual rate of neonates receiving TH in each cooling centre varied between 3 and 12 neonates. In only 13% of the analysed hospital years was the annual rate of neonates receiving TH equal to or more than 12. For 19 out of the 20 cooling centres, detailed information on the place of birth was available. Out of these 1,390 neonates, 46% (n=637) were transferred for TH. 4.7% of the transferred neonates were born out-of-hospital, whereas 95.3% (n=607) were born in 111 different hospitals, with a total of 1,298,058 deliveries during the respective data reporting period. Altogether, 55.3%, 18.5%, and 26.2% were born in hospitals caring for high-, medium-, or low-risk pregnancies, respectively. For each hospital, the respective rate of neonates with TH per 1,000 deliveries was calculated and showed variations between different hospitals. However, the median rate was similar among hospitals caring for high-, medium-, or low-risk pregnancies. Our findings could be used for subsequent planning of perinatal care. Since the annual number of neonates treated with TH is rather low in the majority of participating cooling centres, more centralisation is needed. Furthermore, the relative rate of newborns requiring TH is similar in hospitals providing care for high-, medium-, or low-risk pregnancies. In order to provide immediate resuscitation to asphyxiated infants, paediatric expertise should be available in each hospital where infants are born.

## Introduction

Perinatal problems can impair fetal oxygenation, leading to perinatal asphyxia. Newborns with perinatal asphyxia often require immediate resuscitation after birth. Appropriate resuscitative interventions can mitigate or even prevent subsequent development of hypoxic ischemic encephalopathy (HIE) 1 . The majority (57%) of neonates with moderate or severe HIE will either die or will have severe neurological disability at the age of 18 months 2 . Currently, the only effective intervention is a very early start of therapeutic hypothermia (TH), which reduces death and neurological disability down to 45% and improves long-term outcomes 2 3 .

For several neonatal diseases with a high risk of death or long-term sequelae, such as extreme prematurity, it is well known that delivery in a dedicated tertiary care centre reduces mortality and morbidity 4 5 6 . Data suggest that the experience of the care team is an important determinant of outcome 7 8 . Thus, centralization of care is recommended for these high-risk pregnancies and neonates. Centralization of specialised care is usually accompanied by the requirement to treat at least a minimal number of neonates with a certain condition per year in order to ensure the expertise of the care team 7 .

For newborns with perinatal asphyxia, there is limited evidence to make any recommendations for an appropriate health care structure. Recently, German data from a comprehensive nationwide study found increased odds for adverse outcomes in neonates with perinatal asphyxia who were transferred to another facility within 24 hours 9 . Adverse outcomes in transferred neonates could be explained by inappropriate postnatal resuscitation, inadequate neonatal transfer, or delayed start of TH.

Currently, perinatal care in Germany is stratified according to prenatally known risks. Women with a high risk of neonatal complications, such as extreme prematurity or severe congenital abnormality, should deliver in a hospital with a neonatologist present 24/7. If there is only a minor prenatal risk, such as late preterm delivery or minor congenital abnormalities, women should deliver in a hospital with paediatric departments, but no immediate special neonatal expertise is required. Finally, if there is no prenatally known risk, women may deliver in birth hospitals without any immediate availability of paediatric or neonatal expertise. In these hospitals, neonates are resuscitated in case of an emergency by a midwife, obstetrician, or anaesthesiologist. Whereas the stratification is appropriate for prenatally known risks, there is no data available as to whether that stratification also predicts the occurrence of perinatal asphyxia.

In order to close that knowledge gap, we analysed data of neonates receiving TH, since TH is the strongest proxy for neonates with moderate or severe HIE caused by perinatal asphyxia. Centres performing TH provided data in order to analyse the number of infants with TH born within each cooling centre and how many patients were transferred for TH. Secondly, the level of care where transferred neonates were born was analysed. Finally, the rate of neonates with TH was calculated per 1,000 deliveries for each hospital in order to estimate the risk for moderate to severe HIE in relation to prenatally known risk.

## Methods

Members of the DGPM perinatal research collaborative were contacted if their hospital offers TH for newborns. If the contacted centre offered TH and responded, it was considered a “cooling centre” for the purpose of the present analysis – regardless of the number of infants treated per year. The cooling centres accessed their hospital databases in order to detect neonates with TH by using the OPS code for TH (8–607.0) as the selection criteria. Neonates with TH were excluded if they were born below 36 weeks of gestation or if TH was started after the first day of life. Hospital databases did not provide detailed information on the time of TH initiation during the first day of life or whether TH was initiated during transport. Nevertheless, most cooling centres confirmed that local policy aims to start TH as soon as possible after arrival in the hospital but at least within the first six hours of life. For included patients, the place of birth was determined as either inborn, in-hospital but transferred, or out-of-hospital. For in-hospital but transferred neonates, the level of care of the transferring hospital was provided. Finally, for each cooling centre and referral hospital, the annual number of deliveries was obtained from a public database ( www.nutricia.de ) in order to calculate the percentage of neonates with TH per 1,000 deliveries for each hospital.

Cooling centres provided aggregated information (presented as annual numbers) for subsequent overall analysis. There were no further outcome data available for subsequent detailed analysis. Final data are presented as median and range or absolute numbers, as indicated.

## Results

### The annual number of neonates receiving TH shows large inter- and intra-centre differences

Altogether, data for 1,431 neonates with therapeutic hypothermia was obtained from 20 cooling centres. A total of 45% of these cooling centres were able to provide data for the years 2010–2023. The remaining centres provided data for 13 (n=1), 12 (n=1), 11 (n=3), 10 (n=3), 9 (n=1), 6 (n=1) or 4 (n=1) years. During the respective years of data collection, the cooling centres reported a total of 512,913 deliveries. During that time, the average annual delivery rate in each of the 20 cooling centres was a median of 2,156 (range 1,257–3,371).

**Fig. 1:**
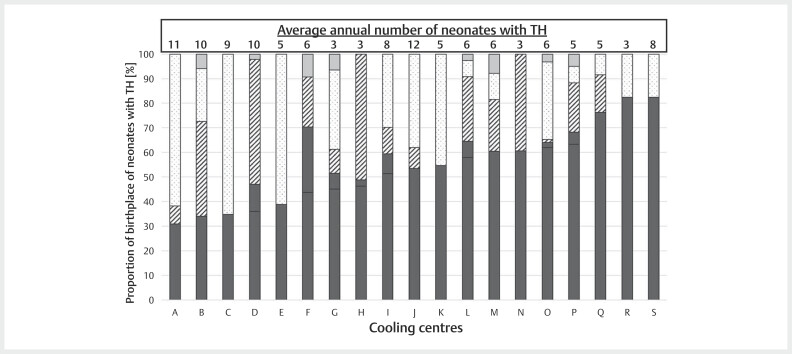
Shown is the relative distribution of the respective birthplace of neonates treated with TH in each cooling centre. Neonates were born in either hospitals caring for high-risk (solid black), medium-risk (stripes), or low-risk pregnancies (dotted) or were born out-of-hospital (grey). Furthermore, the average annual number of infants with TH is shown for each cooling centre.

**Fig. 2:**
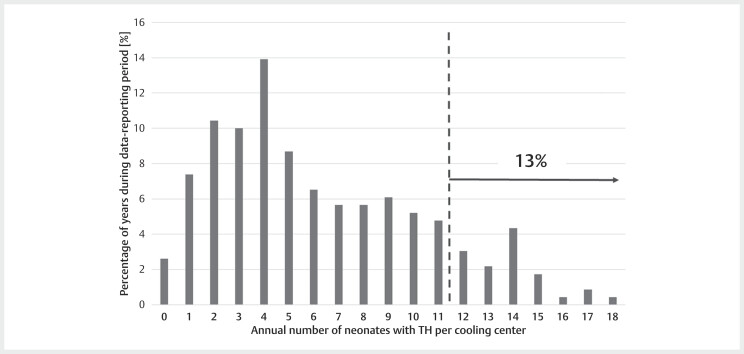
Shown is the relative distribution of annual numbers of neonates receiving TH in each cooling centre for the reported time period. In only 13% of the analysed years, 12 or more patients were treated with TH per year and cooling centre (dotted vertical line).

The average annual rate of neonates receiving TH during the respective data reporting period in each cooling centre varied between 3 and 12 neonates ( Fig. 1 ). Analysis of the respective data reporting period revealed that in only 13% of the analysed hospital years, the annual rate of neonates receiving TH was equal to or more than 12 ( Fig. 2 ).

### A substantial proportion of neonates was transferred from other places of birth

A total of 19 out of the 20 cooling centres provided detailed information on the place of birth of neonates with TH, allowing further analysis. These 19 cooling centres treated a total of 1,390 neonates with TH, with 46% (n=637) being transferred. In two centres, more than 80% of neonates with TH were inborn, whereas three centres had a rate of less than 35% inborn neonates ( Fig. 1 ). Thirty of the transferred neonates (4.7%) were born out-of-hospital, whereas 95.3% (n=607) were born in 111 different hospitals, with a total of 1,298,058 deliveries during the respective data reporting period.

### Relative percentage of neonates receiving TH is similar regardless of pregnancy risk

**Fig. 3:**
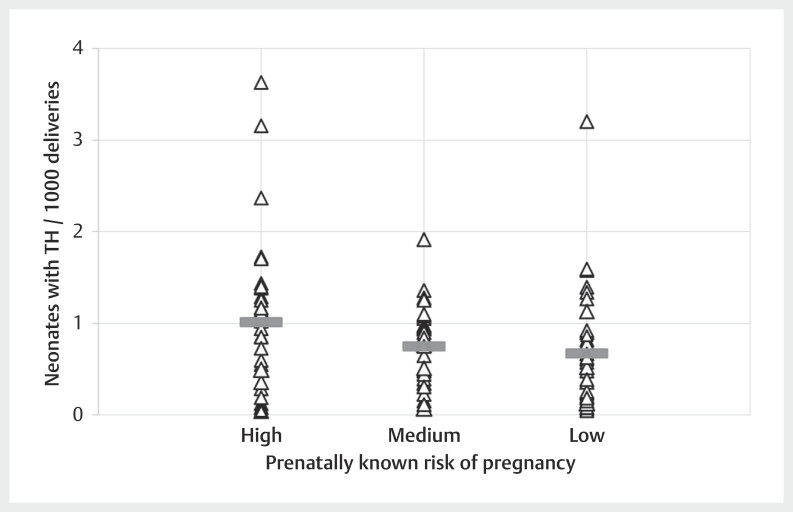
Shown is the average rate of neonates with TH per 1,000 deliveries (triangles). The average rate for the respective data reporting period was calculated for each hospital where a neonate was born and received TH. Furthermore, the median of the average rates of neonates with TH per 1,000 deliveries is shown as a thick grey bar for hospitals with low-, medium-, and high-risk pregnancies.

Analysis of all available data of in-hospital born neonates with TH (n=1,360) revealed that 55.3%, 18.5% and 26.2% were born in hospitals caring for high-, medium-, or low-risk pregnancies, respectively. At the same time, 50.6%, 19.1%, and 30.1% of all deliveries in the 131 hospitals took place in hospitals caring for high-, medium-, or low-risk pregnancies, respectively. Based on the annual number of deliveries in each hospital, the respective rate of neonates with TH per 1,000 deliveries was calculated. Whilst the relative percentage showed large variations between different hospitals, the median of neonates with TH per 1,000 deliveries was similar among hospitals caring for either high-, medium-, or low-risk pregnancies ( Fig. 3 ).

## Discussion

Our data provide important and novel information. Firstly, the annual number of neonates treated with TH is rather low in the majority of participating cooling centres. Secondly, about 46% of all neonates were out-born and subsequently transferred to the cooling centre for TH. Finally, the relative rate of newborns requiring TH is similar in hospitals providing care for high-, medium-, or low-risk pregnancies. These findings have a significant impact and should be used to organize perinatal care in Germany.

Perinatal asphyxia necessitates immediate medical interventions, including advanced resuscitation of the newborn. The quality of neonatal resuscitation has a significant impact on survival and morbidity of affected neonates. Whereas immediate response to resuscitation can prevent subsequent damage, prolonged periods of insufficient cardiorespiratory activity increase the likelihood of severe HIE 1 . Currently, the only intervention to reduce neurological long-term sequelae in moderate or severe HIE is an early start of TH 2 10 . Previous data from Germany and other countries have shown that late start of TH, transfer to a cooling centre within the first 24 hours of life or inappropriate cooling strategies during transport increase the risk of poor outcome 9 10 11 . Besides the great individual burden of subsequent long-term sequelae, subsequent health care and other associated costs equal approximately three million euros per affected child 12 . In order to prevent problems for neonates and affected families and to reduce societal costs, efforts should focus on making immediate neonatal expertise available for every asphyxiated newborn. The present data, however, show room for improvement for the German health care system.

Our data revealed that the relative rate of neonates with moderate or severe HIE is similar in hospitals providing care for low, medium or high-risk pregnancies. This data verifies that complications leading to perinatal asphyxia cannot be predicted prenatally, and thus neonatal or at least paediatric expertise should be available for every delivery. For the attending paediatrician expertise and regular training in neonatal resuscitation is warranted. Furthermore, a paediatrician in a small hospital with limited experience in neonatal resuscitation could benefit from neonatal support via telemedicine 13 14 . Deliveries, even of low-risk pregnancies, without the immediate availability of a paediatrician should be avoided in order to reduce the risk of mortality or severe long-term morbidity.

Recent data from a comprehensive nationwide study found increased odds for adverse outcomes in neonates with perinatal asphyxia who were transferred to another facility 9 . These findings together with our data support the concept that neonatal expertise should be available in every hospital where a baby is born to ensure immediate neonatal resuscitation. Furthermore, a standardized approach is required on how an experienced neonatal care team can be involved immediately in order to ensure high-quality transfer to an experienced cooling centre with appropriate cooling strategies during transport and timely initiation of TH 11 .

For several neonatal diseases, the association between annual number of patients with a certain problem, expertise of health care providers, and subsequent outcome is well documented. Our data, however, show that in 87% of the analysed years, the cooling centres treated less than one neonate per month. Thus, centralization of neonates receiving TH should be further improved.

One conclusion is that regional perinatal networks are urgently needed, as recently suggested by an expert commission from the German Ministry of Health 15 . For a certain region, one specialized cooling centre should provide not only neonatal expertise for resuscitation via telemedicine for paediatricians in regional hospitals but also a dedicated transport service. Assuming that at least one neonate with TH per month is needed to maintain the expertise and knowing that one to two neonates per 1,000 deliveries require TH, every cooling centre should cover a region of at least 10,000 deliveries.

Whereas our data are of great interest, some limitations have to be discussed. Firstly, our rate of moderate or severe HIE is slightly lower than previously described for other countries 16 . That finding can be either explained by better antenatal and perinatal care in Germany or an underdetection of neonates with moderate or severe HIE. Secondly, we did expect that in hospitals caring for high-risk pregnancies the odds of neonates with moderate or severe HIE are much higher – an assumption that was not supported by our findings. It could be speculated that in these hospitals, special obstetric expertise reduces the occurrence of asphyxia and health care providers are more experienced in resuscitation of asphyxiated neonates, which subsequently reduces the risk of HIE and the need for TH. Thirdly, our data is based on a selected number of hospitals in Germany. In the cooling centres and transferring hospitals, a total of 596,116 neonates were born between 2020–2023, equalling about 21% of all deliveries in Germany during that time period. Furthermore, our study covered data from nine different federal states, suggesting that our findings are representative for Germany. Finally, generalisability of our results is limited since our study neither included all neonates with asphyxia nor reported on outcome data. Therefore, it is of importance to include neonates with perinatal asphyxia and TH in registries, as suggested previously and currently performed 17 18 19 .

In conclusion, the present data are another piece of a puzzle suggesting that obstetrics should be performed only in appropriately equipped hospitals. Recently, attempts have been made to establish advanced birth centres in the USA for economic reasons and to reduce the pressure on care providers 20 . However, it was finally warned that “advanced birth centres would be inappropriate and suboptimal sites for pregnant patients to receive care and, unfortunately, they will do nothing to address the maternity care access.” A similar warning is true if obstetrics is performed without immediate availability of paediatric support. The more than 600 neonates suffering every year from severe consequences of perinatal asphyxia in Germany demand regional perinatal care with telemedicine support. Appropriate structures have been recently suggested for Germany and good examples are already established 15 21 . If long-term outcomes could be improved in only 10% of affected neonates, more than 150 million euros of subsequent costs would be saved every year 12 . Thus, there would be an immediate return on the financial investment needed to provide regional perinatal care structures.

**DGPM Perinatal Research Collaborative**

Eva Mildenberger ^6^ , Kirsten Glaser ^7^ , Thorsten Orlikowsky ^8^ , Ursula Felderhoff-Müser ^9^ , Thomas Höhn ^10^ , Claudia Roll ^11^ , Andreas Wemhöner ^12^ , Constantin von Kaisenberg ^13^ , Ekkehard Schleussner ^14^ , Guido Stichtenoth ^15^ , Holm Schneider ^16^ , Zana Aliu Miftari ^17^ , Florian Guthmann ^18^ , Patrick Neuberger ^19^ , Christian Gille ^20^ , Robby Wießner ^1^

^1^ Saxonian Center for Feto/Neonatal Health, Faculty of Medicine and University Hospital Carl Gustav Carus, Technische Universität Dresden, Dresden, German Center for Child and Adolescent Health (DZKJ), SaxoChild partner site Dresden/Leipzig, Germany

^2^ Department of Obstetrics and Gynecology, LMU University Hospital, LMU Munich, Germany

^3^ Department of Neonatology and Interdisciplinary Centre for Cleft Palate and Craniofacial Malformations, University of Tuebingen, Tuebingen, Germany

^4^ University of Cologne, Faculty of Medicine and University Hospital Cologne, Department of Pediatrics, Division of Neonatology, Cologne, Germany

^5^ Department of Obstetrics, University of Würzburg, Würzburg, Germany

^6^ Division of Neonatology, Department of Pediatrics, University Medical Center of the Johannes Gutenberg University Mainz, Mainz, Germany

^7^ Division of Neonatology, Department of Women's and Children's Health, University of Leipzig Medical Center, Leipzig, Germany

^8^ Department of Pediatrics, Faculty of Medicine, RWTH Aachen University, Aachen, Germany.

^9^ Department of Pediatrics I (Neonatology, Ped. Intensive Care, Ped. Infectiology, Ped. Neurology), University Hospital Essen, Center of Translational Neurobehavioural Sciences C-TNBS, University of Duisburg-Essen, Essen, Germany

^10^ Department of Neonatology, Medical Faculty, University Medicine Düsseldorf, Düsseldorf, Germany

^11^ Department of Neonatology, Pediatric Intensive Care, Sleep Medicine, Vest Children's Hospital Datteln, University Witten-Herdecke, Datteln, Germany

^12^ Department of Neonatology and Pediatrics, Marienhospital Gelsenkirchen, Academic Hospital of the Ruhr University Bochum, Gelsenkirchen, Germany

^13^ Department of Obstetrics, Gynecology and Reproductive Medicine, Hanover Medical School, Hanover, Germany

^14^ Department of Obstetrics, University Hospital Jena, Jena, Germany

^15^ Department of Pediatrics, University Hospital of Lübeck, 23538 Lübeck, Germany

^16^ Neonatologie, Universitätsklinikum Erlangen, Kinder- und Jugendklinik, Erlangen, Germany

^17^ Klinik St. Hedwig, Lehrstuhl für Frauenheilkunde und Geburtshilfe (Schwerpunkt Geburtshilfe) der Universität Regensburg, Regensburg, Germany

^18^ Neonatologie, AUF DER BULT, Kinder- und Jugendkrankenhaus, Hannover, Germany

^19^ Department for Neonatology, Klinikum Stuttgart Olgahospital Women's Clinic, Stuttgart, Baden-Württemberg, Germany

^20^ Department for Neonatology, Center for Child and Adolescent Medicine, Heidelberg University Hospital, Heidelberg, Germany
